# Supplementary material for: Optogenetic dissection of transcriptional repression in a multicellular organism
Source: Nat Commun. 2024 Oct 26;15:9263. doi: 10.1038/s41467-024-53539-0 (PMC11513125; doi:10.1038/s41467-024-53539-0)
Supplement: Supplementary file 1 — Supplementary Information [file 41467_2024_53539_MOESM1_ESM.pdf]

Supplementary Information

Optogenetic dissection of transcriptional repression in a multicellular organism

Jiaxi Zhao[1,2†], Nicholas C. Lammers[3,4†], Simon Alamos[5,6], Yang Joon Kim[3], Gabriella Martini[7], Hernan G. Garcia[1,3,7,8,9*]

[1]Department of Physics, University of California, Berkeley, CA

[2]Current address: Department of Genetics, Harvard Medical School, Boston, MA; Department of Pathology, Brigham and Women's Hospital, Boston, MA

[3]Biophysics Graduate Group, University of California, Berkeley, CA

[4]Current address: Department of Genome Sciences, University of Washington, Seattle, WA;

[5]Department of Plant and Microbial Biology, University of California, Berkeley, CA

[6]Current address: Feedstocks Division, Joint BioEnergy Institute, Emeryville, CA; Environmental Genomics and Systems Biology Division, LBNL, Berkeley, CA

[7]Department of Molecular and Cell Biology, University of California, Berkeley, CA

[8]Institute for Quantitative Biosciences-QB3, University of California, Berkeley, CA

[9]Chan Zuckerberg Biohub, San Francisco, CA

[†]These authors contributed equally.

[*]To whom correspondence should be addressed: hggarcia@berkeley.edu

Supplementary Notes

1 Additional cpHMM inference results

In this section, we briefly describe additional cpHMM inference results. In addition to the binary inference results shown in Main Text Fig. 4C that examine burst parameter values at high and low Knirps values, we also conducted finer-grained cpHMM inference runs, in which we queried burst parameter values across the full range of Knirps concentrations observed in our experiments. The plots in Supplementary Fig. 11 summarize our results. As with the results in the main text, this inference was conducted on 15-minute-long fragments of transcriptional traces. Multiple such fragments were generated from each transcription trace by sliding a 15-minute window along each and sampling in 1 minute increments. This produced a dataset of transcriptional "reads" that were then grouped by average Knirps concentration. In addition, we grouped transcriptional reads by experiment type (as defined in Main Text Fig. 2B and D): no light (circles in Supplementary Fig. 11), low intensity (diamonds), and high intensity (squares).

We find that the inference results are consistent with the trends indicated in Main Text Fig. 4C. We once again see that the burst frequency decreases with increasing Knirps concentration, though it is notable that the increased dynamic range of our inference reveals a more dramatic dependency, with burst frequency (k_{on}) dropping by a factor of 6 across the range of concentrations examined (Supplementary Fig. 11A). Additionally, we see that the burst duration ($1/k_{\text{off}}$) increases with increasing Knirps and that burst amplitude (r) remains roughly constant (Supplementary Fig. 11B and C). We note that, on its own, the Knirps-dependent increase in burst duration would actually lead to *activation*. Thus, although the burst duration exhibits Knirps-dependence, the burst frequency is the only parameter that is modulated in a manner consistent with the reduction in transcription as a result of repressor action.

However, while these findings paint a more detailed picture of how Knirps regulates transcriptional dynamics than the binary results presented in the main text, their resolution is nonetheless still limited by the fact that we must use 15-minute fragments for cpHMM inference. As a result, this approach is not suitable for recovering the true, instantaneous input-output functions that dictate how Knirps dictates burst parameter values. To make progress toward this goal, we developed a simulation-based computational framework for input-output function inference. We provide further details on this approach in the following sections.

2 Stochastic input-output simulations

Here we provide further details regarding the implementation of the simulation-based computational method that was utilized to produce the results featured in Main Text Fig. 4F-H of the main text. Our aims in developing this method were two-fold: first, we sought to use our live imaging data to uncover burst parameter input-output functions and, second, we sought to assess whether a simple two-state model of transcriptional control based on our inference results in Main Text Fig. 4C is sufficient to explain both the sharp input-output function (Main Text Fig. 2D) and rapid reactivation dynamics (Main Text Fig. 3D-E) revealed by our experiments.

2.1 Model specification

Our coarse-grained cpHMM burst inference results indicate that both burst frequency (k_{on}) and burst duration ($1/k_{\text{off}}$) vary as functions of Knirps concentration (Main Text Fig. 4C). Accordingly, we employed a modeling framework in which both of these parameters vary as a function of Knirps concentration. Specifically, we model k_{on} and k_{off} as simple Hill functions of nuclear Knirps concentration (see inset panel of Main Text Fig. 4F), such that:

$$k_{\text{on}}([\text{Knirps}]) = k_{\text{on}}^0 \frac{K_{D_{on}}^{H_{on}}}{[\text{Knirps}]^{H_{on}} + K_{D_{on}}^{H_{on}}}, \tag{1}$$

and

$$k_{\text{off}}([\text{Knirps}]) = k_{\text{off}}^0 \frac{K_{D_{off}}^{H_{off}}}{[\text{Knirps}]^{H_{off}} + K_{D_{off}}^{H_{off}}}. \tag{2}$$

where k_{on}^0 and k_{off}^0 set the upper limits for on and off rates, respectively; where the Hill coefficient H_{on} and H_{off} set the sharpness of each parameter's response to increasing Knirps concentration; and where $K_{D_{on}}$ and $K_{D_{off}}$ dictate the half-max points for the k_{on} and k_{off} input-output curves. Finally, we assume that the burst amplitude, r, takes on a fixed value that does not vary as a function of Knirps concentration.

2.2 Stochastic simulations

To simulate promoter trajectories with concentration-dependent burst parameters, we used a discrete implementation of the widely used Gillespie Algorithm [1], in which the promoter state is sampled with a time resolution of 1 second. We provide a brief overview of the approach here, and direct readers to the Github repository accompanying this work for further details regarding the algorithm's implementation. Consider the time-varying burst parameter trends shown in Supplementary Fig. 12B, along with the simulated ON/OFF promoter trajectory in Supplementary Fig. 12C. At 11 minutes, we see that the promoter switches into the OFF state. In a standard Gillespie simulation with constant burst parameters, we would obtain the time until the next transition, τ_{OFF}, by drawing a random sample from an exponential distribution with rate parameter $\lambda = k_{\text{on}}$, such that

$$\tau_{\text{OFF}} \sim \text{Exp}(k_{\text{on}}). \tag{3}$$

At time $11 + \tau_{\text{OFF}}$, the promoter would then transition out of the OFF state and into the ON state.

Our case is more complicated, however, since k_{on} may change over time as the nuclear Knirps concentration changes. One simple way to capture this time-dependence is to adopt a discrete approach to promoter state simulations. In this approach, we designate some finite simulation time resolution, Δt. Starting again at $t = 11$ minutes (with the promoter in the OFF state), the algorithm proceeds as follows:

1. Use Equation 1 to calculate k_{on} based off of the current Knirps concentration

2. Sample an expected jump time τ

   if promoter is OFF, sample τ from an exponential distribution with rate parameter k_{on}

   else, sample τ from an exponential distribution with rate parameter k_{off}

3. Compare τ to Δt

   if $\tau \geq \Delta t$: the promoter state remains unchanged

   else, if $\tau < \Delta t$: change the promoter state (OFF to ON in our case)

4. Increment the time variable such that $t = 11 + \Delta t$, and return to (1).

To understand why this discrete rejection procedure for sampling the jump time τ is valid, consider the probability that the promoter remains in the OFF state for longer than n time steps ($P(\tau_{\text{OFF}} > n\Delta t)$). If we were sampling τ_{OFF} directly from the exponential distribution—as is the case for the standard Gillespie Algorithm—the probability of this outcome would be given by:

$$P(\tau_{\text{OFF}} > n\Delta t) = e^{-nk_{\text{on}}\Delta t}. \tag{4}$$

In our discrete rejection-based approach, the probability that $\tau_{\text{OFF}} > n\Delta t$ is given by the joint probability that independently sampled values of τ, drawn at each iteration, are less than the sampling time resolution Δt. The fact that each sample is independent means that the joint probability takes the form of a product:

$$P_{\text{samp}}(\tau_{\text{OFF}} > n\Delta t) = P(\tau_{\text{OFF}} > \Delta t)_{t_1} \times P(\tau_{\text{OFF}} > \Delta t)_{t_2} \times \ ...\ P(\tau_{\text{OFF}} > \Delta t)_{t_n}. \tag{5}$$

Simplifying, we see that the discretely sampled probability exactly equals the true probability

$$P_{\text{discrete}}(\tau_{\text{OFF}} > n\Delta t) = \left[P(\tau_{\text{OFF}} > \Delta t)_{t_1}\right]^n = e^{-nk_{\text{on}}\Delta t}. \tag{6}$$

The main advantage of our discrete approach relative to the standard Gillespie Algorithm is that we are able to change the rate parameter (k_{on} or k_{off}) at each sampling step to reflect changing Knirps concentrations. This leads to sampled jump time distributions of the form:

$$P_{\text{samp}}(\tau_{\text{OFF}} > n\Delta t) = e^{-k_{\text{on}}\left([Knirps]_{t_0}\right)} \times e^{-k_{\text{on}}\left([Knirps]_{t_1}\right)} \times \ ...\ e^{-k_{\text{on}}\left([Knirps]_{t_n}\right)} \tag{7}$$

that reflect the effects of dynamic transcription factor concentrations.

Thus, by following our discrete sampling procedure, we obtain a discrete time trace of promoter activity, $\boldsymbol{p}(t)$, that reflects time-dependent changes to the transition rates k_{on} and k_{off} due to changes in Knirps concentration. We set $\Delta t = 1$ second, such that the resolution of our discrete sampling is significantly faster than the promoter burst dynamics being simulated (defined by k_{on} and k_{off}; see Main Text Fig. 4C). By enforcing this separation of timescales, we ensure that our discretely sampled time trace is a good approximation of a continuous Knirps-dependent trajectory.

Unlike k_{on} and k_{off}, we assume that the initiation rates, r_0 and r_1, which encode the rate of Pol II initiation in the OFF and ON states, respectively, are Knirps-independent. Note that, for simplicity, we refer to r_1 simply as "r" in the main text, and do not discuss results for r_0 since $r_0 \approx 0$. Thus, to obtain a predicted time series of initiation rates, $\boldsymbol{r}$ from promoter states $\boldsymbol{p}$, we simply, set $\boldsymbol{r} = r_0$ for all time points when the promoter is OFF and $\boldsymbol{r} = r_1$ for all time points when the promoter is ON (see inset panel of Supplementary Fig. 12C). Finally, we obtain a predicted MS2 trace shown in Supplementary Fig. 12D by convolving $\boldsymbol{r}$ with the kernel κ_{MS2} Supplementary Fig. 12D, inset), which has the effect of taking a moving sum of past initiation rates over a time window defined by the time required for which nascent polymerase molecules remain on the gene body (set to 140 seconds throughout this work). This procedure also accounts for the finite amount of time needed for newly initiated Pol II molecules to transcribe the MS2 cassette and become fluorescent. We direct readers to Appendix D of [2] for further details.

2.3 Parameter sweeps

We used parameter sweeps to systematically test model performance across a broad range of plausible parameter values. As illustrated in Supplementary Fig. 12E, we performed a gridded sweep across 15 different values for $K_{D_{on}}$ and H_{on} from Equation 1. In addition we sampled 15 values each for $K_{D_{off}}$ and H_{off} (not pictured) from Equation 2, making for a total of $15^4 = 60625$ distinct parameter combinations. The remaining parameters, namely k^0_{on}, k^0_{off}, r_0, and r_1 were held fixed at their average values as calculated from the Knirps-dependent inference results shown in Supplementary Fig. 11A-C. Table 3 specifies the values and value ranges used for this procedure.

For each combination of parameter values, the procedure outlined in Supplementary Fig. 12A-D was used to generate ensembles of simulated fluorescent traces with realistic Knirps-dependent burst parameters using real experimental measurements of Knirps concentration over time (Supplementary Fig. 12F). We could then use these trace ensembles to calculate predictions for the fluorescence vs. [Knirps] input-output function and reactivation cumulative distribution function (CDF, Supplementary Fig. 12G). By comparing our model predictions to our experimental results (Supplementary Fig. 12G), it was possible to assess whether a given set of model parameters was sufficient to recapitulate these key features of Knirps repression.

We used the mean-squared error to assess model fits to the input-output function and reactivation CDF. In each case, deviations were normalized by the mean of the experimental curve to ensure comparable scaling between the fluorescence input-output errors (which are natively in arbitrary units) and CDF errors (which are probabilities). For the fluorescent input-output function (Main Text Fig. 4G) this gives

$$\delta^2_{io} = \frac{1}{N_k} \sum_{k=1}^{N_k} \left(\frac{f_k - \hat{f}_k}{\mu_f} \right)^2, \tag{8}$$

where N_k is the number of Knirps concentration bins for which the average was calculated, μ_f is the average fluorescence of the experimental curve in Main Text Fig. 4G taken across all N_k points, and where f_k and $\hat{f}_k$ are the observed and predicted fluorescent values for Knirps concentration group k. Similarly, for the reactivation CDF we have

$$\delta_{ra}^2 = \frac{1}{N_t}\sum_{k=1}^{N_t}\left(\frac{p_t - \hat{p}_t}{\mu_p}\right)^2, \tag{9}$$

where N_t is the number of time points post-reactivation that were considered, μ_p is the average probability taken across the CDF in Main Text Fig. 4H, and where p_t and $\hat{p}_t$ are the observed and predicted fraction of reactivated nuclei at time point t post Knirps export.

We defined the total error in model fit as the weighted sum of δ_{io}^2 and δ_{ra}^2, such that

$$\delta^2 = (w_{io}\delta_{io}^2 + w_{ra}\delta_{ra}^2)(N_k + N_t), \tag{10}$$

where the sum $(N_k + N_t)$ up-weights δ^2 according to the total number of data points considered, and where w_{io} and w_{ra} are weight parameters that tune the relative impact of δ_{io}^2 and δ_{ra}^2 to the total loss, δ^2. These weights can be adjusted to navigate tradeoffs between the minimization of input-output and reactivation CDF fitting loss. In our case, we find that values of $w_{io} = 1/4$ and $w_{ra} = 3/4$ lead to the best visual alignment between model predictions and experimental observations.

2.4 Estimating uncertainty bounds with MCMC

The parameter sweep procedure outlined above produced a δ^2 estimate for each of the 60625 parameter combinations considered. In principle, the model realization corresponding to the lowest δ_t^2 could be selected to obtain an approximate point estimate for the optimal $K_{D_{on}}$, H_{on}, $K_{D_{off}}$, and H_{off} values; however the parameter sweep results are not alone sufficient to obtain uncertainty bounds, nor do they provide insights into the remaining parameters not included in the sweep. To obtain this information, we employed Markov Chain Monte Carlo (MCMC) to sample the posterior distributions of our model parameters, conditional on our experimental data. MCMC is a widely used class of algorithms that are capable of efficiently sampling high-dimensional probability distributions [3].

As a first step in this process, we utilize information from the parameter sweeps to obtain parameter priors that are used to initialize and constrain MCMC sampling. To do this, we generate a weight vector, $\boldsymbol{w}$, comprised of terms with the form

$$w_i = e^{-\delta_i^2}, \tag{11}$$

where δ_i^2 is the total loss from Equation 10 for the ith set of parameter values. If we assume that model errors are approximately Gaussian-distributed, then each w_i can be interpreted as an unnormalized probability that is proportional to the likelihood of the data $\boldsymbol{x}$ (the input-output and reactivation curves) conditional on the ith parameter set $\boldsymbol{\theta_i}$:

$$w_i \propto P(\boldsymbol{x}|\boldsymbol{\theta_i}). \tag{12}$$

Moreover, from Bayes' Theorem we have that

$$w_i \propto P(\boldsymbol{x}|\boldsymbol{\theta_i})P(\boldsymbol{\theta_i}) = P(\boldsymbol{\theta_i}|\boldsymbol{x})P(\boldsymbol{x}). \tag{13}$$

From here, we see that if we take a uniform prior across all $\boldsymbol{\theta_i}$ values (such that $P(\boldsymbol{\theta_i})$ is a constant), then the weight w_i will be proportional to the likelihood of each set of parameter values, conditional on the experimental data:

$$w_i \propto P(\boldsymbol{\theta_i}|\boldsymbol{x}). \tag{14}$$

Motivated by this observation, we resampled the parameter values, $\boldsymbol{\theta}$, surveyed in the parameter sweep according to the weight vector $\boldsymbol{w}$. This leads to a new set of parameter values, $\boldsymbol{\theta}^*$, where the frequency of a given parameter vector, $\boldsymbol{\theta_i}$, is proportional to its likelihood. As a result, the best-fitting parameter sets will appear more frequently in $\boldsymbol{\theta}^*$, and the worst-fitting are unlikely to appear at all. We calculate prior distributions for $K_{D_{on}}$, H_{on}, $K_{D_{off}}$, and H_{off} (assumed to be Gaussian) by taking the mean and standard deviation of each parameters values across $\boldsymbol{\theta}^*$. The prior distributions for k^0_{on}, k^0_{off}, and r_1 were initialized using the Knirps-dependent cpHMM inference results shown in Supplementary Fig. 11A-C. Specifically, the mean and standard deviation for k^0_{on} and k^0_{off} were estimated using the mean and standard deviations of the intercepts of the linear fits shown in Supplementary Fig. 11A and B, which we reasoned should provide reasonable estimates for the upper limit of each parameter. Given the lack of strong Knirps-dependence in the burst amplitude, the mean and standard deviation for the r_1 prior were calculated by taking the mean and standard deviation of all cpHMM results shown in Supplementary Fig. 11C. The initiation rate when the system is in the OFF state, r_0, was not subject to MCMC sampling, and was held fixed at its mean value from cpHMM inference. See Table 4 for the precise values used for each parameter prior.

With our prior distributions established, we conducted MCMC sampling to obtain estimates for the posterior distribution of each parameter. We conducted 24 independent MCMC simulations, each of which was run for 2500 total steps. We used standard Metropolis Hastings [4] updates during sampling. The procedure for each step was as follows:

1. At the tth step in the simulation, a new proposal for the parameter vector, $\boldsymbol{\theta'_t}$, was generated by sampling from a multivariate normal distribution centered at the parameter values from the previous step, such that

$$\boldsymbol{\theta'_t} \sim \mathcal{N}(\boldsymbol{\theta_{t-1}}, \boldsymbol{\Sigma}). \tag{15}$$

   The covariance matrix, $\boldsymbol{\Sigma}$, dictates how large or small the randomly proposed jumps tend to be relative to the previous parameter values. We assumed $\boldsymbol{\Sigma}$ to be a diagonal matrix and set each component, σ_i, to be equal to 15% of the standard deviation of the corresponding parameter's prior distribution.

2. Next, we used the proposed parameters, $\boldsymbol{\theta'_t}$, to simulate populations of MS2 traces and calculate predictions for the fluorescence vs. Knirps curve (Main Text Fig. 4G) and reactivation CDF (Main Text Fig. 4H) as outlined in the preceding sections.

3. We then calculated the total likelihood of the new parameters, defined as

$$P(\boldsymbol{\theta'_t}|\boldsymbol{x}) = P(\boldsymbol{x}|\boldsymbol{\theta'_t})P(\boldsymbol{\theta'_t}). \tag{16}$$

Here the first term on the right-hand-side is as defined in Equations 11 and 12, and functions to penalize proposals that produce curves that deviate too far from experimental measurements. The second component is the prior probability, and has the effect of penalizing proposals that deviate too far from our priors regarding parameter values.

4. Finally, we perform the standard Metropolis-Hastings move [4, 5]. We calculate a probability, p, that takes the form

$$p = min\left\{\frac{P(\boldsymbol{\theta'_t}|\boldsymbol{x})}{P(\boldsymbol{\theta_{t-1}}|\boldsymbol{x})}, 1\right\}, \tag{17}$$

where $P(\boldsymbol{\theta_{t-1}}|\boldsymbol{x})$ is the likelihood of the previous set of parameter values. Next we draw a random number, z, from the uniform distribution ($z \sim \mathcal{U}[0,1]$). If $p \geq z$: $\boldsymbol{\theta_t} = \boldsymbol{\theta'_t}$. Otherwise: $\boldsymbol{\theta_t} = \boldsymbol{\theta_{t-1}}$.

2.5 Additional MCMC results

Figure 14 contains bivariate density plots and univariate histograms illustrating the results of MCMC sampling for each of the seven parameters examined. The results for the burst frequency (k_{on}) are as quoted in the main text. We find that, like k_{on}, k_{off} has a negative dependence on ($H_{off} = 3.2 \ \pm 0.65$). This translates to a burst duration that is predicted to *increase* as a function of increasing Knirps concentration (Supplementary Fig. 14C). On its own, this trend would *increase eve* 4+6 activity; however, this effect is dominated by the stronger Knirps-dependent decrease in k_{on}, leading to a strong overall repressive effect (see Main Text Fig. 4G). Additionally, our sampling returns a burst amplitude (r_1) value of $21.6 \ \pm 1.9$ au/min.

3 Implementation of the thermodynamic binding model

Here we provide a brief description of the theoretical underpinnings of the binding model that was used to generate the solid black curve in Main Text Fig. 4F. The core assumption of this model is that k_{on} is inversely proportional to the number of Knirps molecules bound to the locus, such that

$$k_{\text{on}} = k^0_{\text{on}}\left(1 - \frac{n_b}{N}\right), \tag{18}$$

where k^0_{on} is the maximum burst frequency value (set to the 2.8 min^{-1} value returned by MCMC inference), n_b is the number of Knirps molecules bound, and N is the total number of binding sites along the enhancer. Using PATSER scores for the *eve* 4+6 enhancer, we assess that there are 10 Knirps binding sites along the enhancer, such that $N = 10$ (see Supplementary Fig. 6). Thus, in this model $k_{\text{on}} = 0$ when N sites are bound and $k_{\text{on}} = k^0_{\text{on}}$ when 0 sites are bound.

Knirps-dependence enters into Equation 18 through n_b, which should vary as a function of Knirps concentration. Note that, for ease of notation, we denote Knirps concentration by $[R]$ (as opposed to $[Knirps]$) throughout this appendix. To model n_b, we adopt the simple equilibrium chain model developed in [6]. Briefly, this model assumes that all binding sites are identical, such that there are only $N + 1$ distinct binding states in which the enhancer can exist, ranging

from 0 sites bound to all N sites bound. In this model, the Knirps concentration will induce a probability distribution over the set of possible binding states. Each state's probability is given by

$$p(n_b; [R]) = \frac{W(n_b)\omega^{\frac{n_b(n_b-1)}{2}}\left(\frac{K_D}{[R]}\right)^{-n_b}}{Z}, \tag{19}$$

where K_D is the dissociation constant for Knirps binding to specific sites at the locus. We assume that any pair of Knirps molecules can interact with a cooperativity factor ω. Given n_b bound Knirps molecules, there are $\frac{n_b(n_b-1)}{2}$ such pairwise interactions and, hence, a cooperativity contribution of $\omega^{\frac{n_b(n_b-1)}{2}}$. Further, $W(n_b)$ accounts for the number of different microscopic binding configurations that correspond to each macroscopic binding state (i.e., in how many different configurations can n_b Knirps molecules be bound?):

$$W(n_b) = \frac{N!}{(N-n_b)!n_b!}. \tag{20}$$

Lastly, we note that the denominator Z in Equation 19 is a normalizing factor equal to the sum of all $N+1$ numerators. We direct the reader to Appendix C.3 of [6] for a detailed derivation of Equation 19.

We then use this expression for $p(n_b)$ to calculate the average expected number of bound Knirps molecules as a function of nuclear Knirps concentration, such that

$$\big\langle n_b([R])\big\rangle = \sum_{n_b=0}^{N} p(n_b; [R])n_b. \tag{21}$$

Finally, we plug Equation 21 into Equation 18 for the experimentally observed range of Knirps concentrations to produce a predicted burst frequency vs. Knirps input-output curve like the one shown in Main Text Fig. 4F.

Using this approach, we conduct nonlinear least squares fitting to identify the values of ω and K_D that best fit the blue k_{on} trend in Main Text Fig. 4F that was returned by our MCMC inference. Our fit indicates that K_D and ω values of 70.8 au and 1.9, respectively, produce the optimal fit. We find that the k_{on} trend generated by these parameter values (black in Main Text Fig. 4F) is in close agreement with our MCMC inference result (blue curve), demonstrating that simple equilibrium binding could explain Knirps regulation of the burst frequency.

4 Comparison to other optogenetic approaches developed for multicellular organisms

In this work, we build on previous works using the LEXY technology [7–11] and demonstrate the power of the LEXY system for modulating protein dynamics inside developing embryos. The LEXY tag-based method addresses several key limitations faced by many previously reported methods.

First, some optogenetic tools are designed for specific signaling pathways [12–17], and receptor [18] targets, and as a result, are not readily generalizable. In contrast, LEXY can be

directly attached to any protein (though issues of genetic rescue [10] and its modulation strength [8] remain).

Second, many optogenetic tags do not act through concentration modulation, which makes it difficult to draw quantitative conclusions from the results. For example, the blue light-induced dimerization of *Arabidopsis* cryptochrome 2 (CRY2) controls downstream transcription by disrupting the function of the tagged protein through multimerization without affecting its concentration [19–21]. On the other hand, LEXY controls transcriptional activity through direct modulation of the protein concentration within the nucleus, allowing for easy quantification and straightforward interpretation.

5 Frequency-based repression is sub-optimal from a noise perspective

A key finding of this work is that Knirps represses *eve* 4+6 via the down-regulation of the transcriptional burst frequency (k_{on}). It is thus of interest to examine whether frequency-based repression confers desirable performance characteristics to the transcriptional system. One natural perspective from which to ask this question (though by no means the only one) is through the lens of transcriptional noise: does repression via the burst frequency lead to lower noise levels than repression by decreasing burst duration or burst amplitude? In this section we perform a few simple calculations to examine this question.

We use the coefficient of variation (CV) in the transcription rate to quantify noise levels. From [22], we have that the mean transcription rate for the two-state model bursting model (Main Text Fig. 4A) is given by:

$$\mu = r\frac{k_{\text{on}}}{k_{\text{on}} + k_{\text{off}}}, \tag{22}$$

where r is the burst amplitude, k_{on} is the burst frequency, and k_{off} is the inverse of the burst duration. We also have that the variance in the transcription rate is

$$\sigma^2 = r^2\frac{k_{\text{on}}k_{\text{off}}}{(k_{\text{on}} + k_{\text{off}})^3}. \tag{23}$$

Combining Equations 22 and 23, we obtain an expression for the CV:

$$CV = \frac{\sigma}{\mu} = \frac{2}{k_{\text{on}} + k_{\text{off}}}\left(\frac{k_{\text{off}}}{k_{\text{on}}}\right)^{\frac{1}{2}}. \tag{24}$$

The first thing to note is that the CV does not depend on the burst amplitude, r, such that repressing transcription this way is neutral with respect to noise levels. That leaves two more ways to repress transcription: decrease k_{on} or increase k_{off}.

First, consider the k_{off} strategy: in this case k_{off} becomes large while k_{on} is held constant. In the limit where $k_{\text{off}} >> k_{\text{on}}$, this leads to a CV that scales as $(1/k_{\text{off}})^{\frac{1}{2}}$. This implies that noise *decreases* as k_{off} grows larger and the transcription decreases. Conversely, if k_{on} becomes small (the scenario we see in our data), the CV scales as $(1/k_{\text{on}})^{\frac{1}{2}}$. This points to a sub-optimal scenario in which noise *increases* as k_{on} grows smaller and expression decreases.

Thus, of the three burst parameters, repression via the burst frequency exhibits the least desirable noise characteristics. While much more work is needed to connect these simple calculations to the realities of repression *in vivo*, they do nonetheless suggest that the Knirps-*eve* 4+6 system is not optimized for noise reduction.

Supplementary Figures

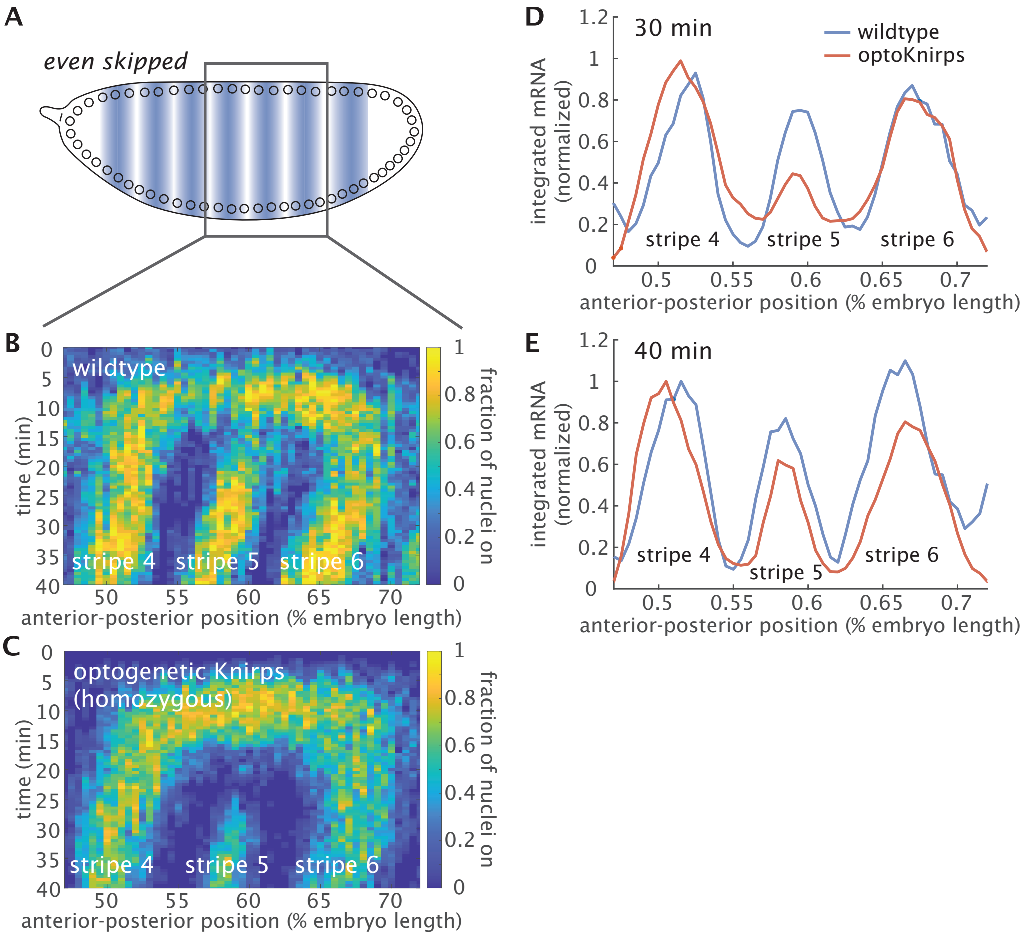

Supplementary Fig. 1: ***even-skipped* expression under homozygous optogenetic Knirps (tagged with LEXY and LlamaTag) qualitatively recapitulates wild-type expression dynamics.** (**A**) To understand whether and to what degree the *eve* expression pattern is impacted in the homozygous optogenetics Knirps background, we imaged the dynamics of a previously published eve-MS2-BAC reporter containing the full endogenous *eve* locus [23] in the wild-type and optogenetics Knirps backgrounds. (**B-C**) The expression pattern of *even-skipped* as reported by the fraction of detectable MS2 transcription spots is similar under wild-type Knirps (B) and optogenetics Knirps (C) except for a weaker stripe 5. (**D-E**) Comparison of the amount of mRNA present at 30 minutes into nuclear cycle 14 (as obtained by integrating the MS2 fluorescence signal) and at 40 minutes shows that stripe 5 expression is weaker under homozygous optogenetics Knirps at 30 minutes. The integration was performed assuming a mRNA half-time of 7 min. (D) Stripe 4 and 6 expression is slightly wider than under the wild-type condition at 30 min, suggesting that optogenetics Knirps is a slightly weaker repressor compared to the wild-type Knirps. (E) Stripe 5 expression continues to increase as it reaches a similar level compared to the wild-type around 40 minutes. The anterior-posterior position is aligned based on the center of stripe 5. The plots are normalized according to the peak of stripe 4 at 40 minutes and smoothened using a moving window of 1.5% range along the anterior-posterior axis. (Data from a single embryo is shown for each condition. $t = 0$ is defined as the onset of transcription in nuclear cycle 14.)

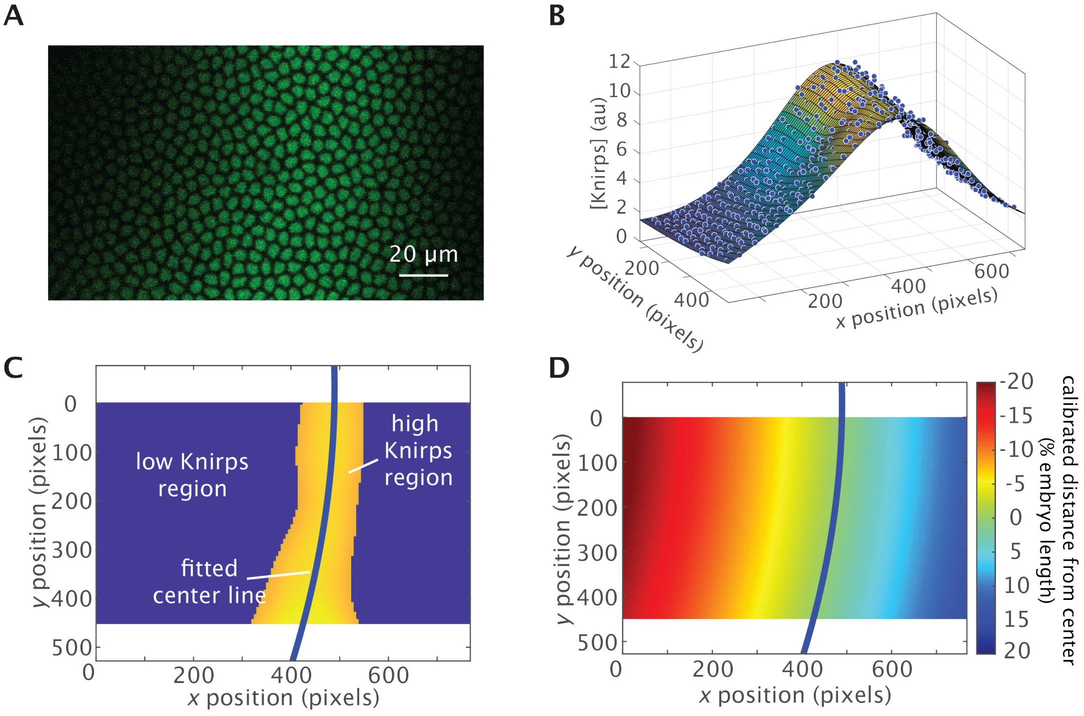

Supplementary Fig. 2: **Nuclei position calibration based on Knirps expression pattern.** The Knirps pattern of each individual embryo is used to align embryos along their anterior-posterior position axis. (**A**) Snapshot of the Knirps pattern used to calibrate nuclei position. (**B**) Extracted nuclear fluorescence is smoothed by local quadratic regression. (**C**) The region with high Knirps expression (yellow region) is extracted with a single threshold. Then, a quadratic function is fitted to the nuclei with high Knirps expression (yellow region) to extract the center line of Knirps expression (blue line). (**D**) Calibrated positions relative to the Knirps expression peak are calculated based on the distance to the extracted center line.

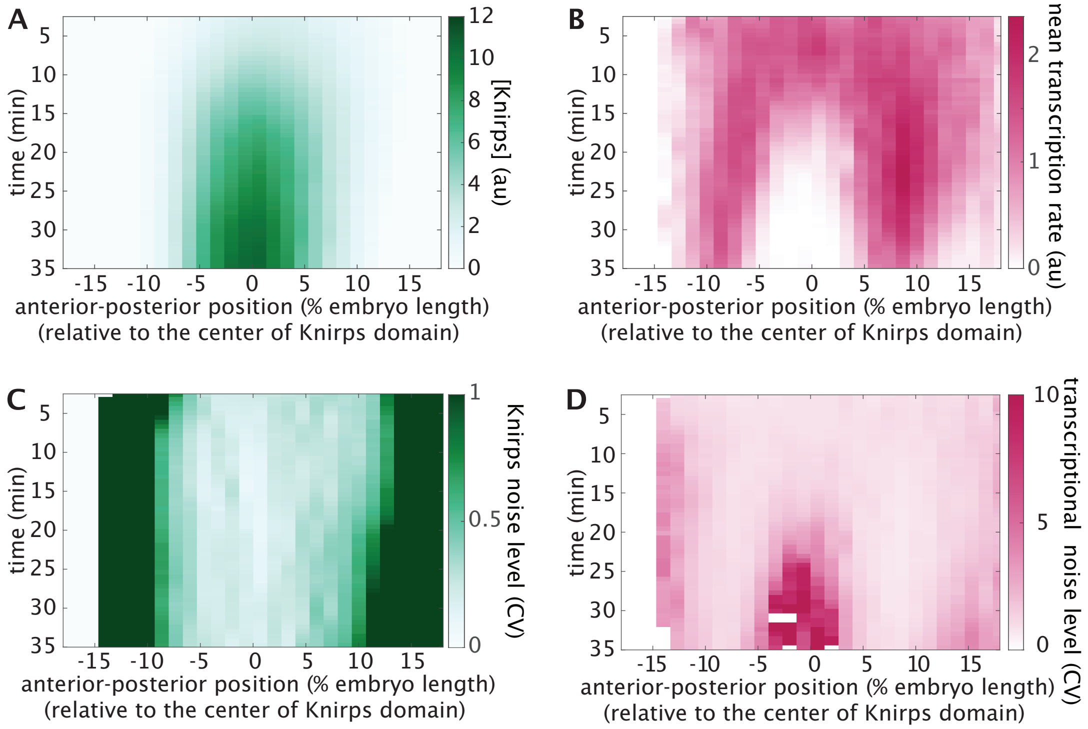

Supplementary Fig. 3: **Spatiotemporal dynamics of Knirps protein and *eve* 4+6 transcription.** Nuclei were binned based on their positions relative to the center of the Knirps domain (Supplementary Fig. 2, Materials and Methods) and their corresponding (A) Knirps protein concentration reported by LlamaTag fluorescence and (B) transcription reported by MS2 fluorescence were quantified over time. Additionally, spatiotemporal patterns in noise levels were quantified using the coefficient of variation (CV). The CV is defined as the standard deviation divided by the mean, and thus captures the magnitude of nucleus-to-nucleus variability relative to the mean in (C) in Knirps protein levels and (D) *eve* 4+6 transcription.

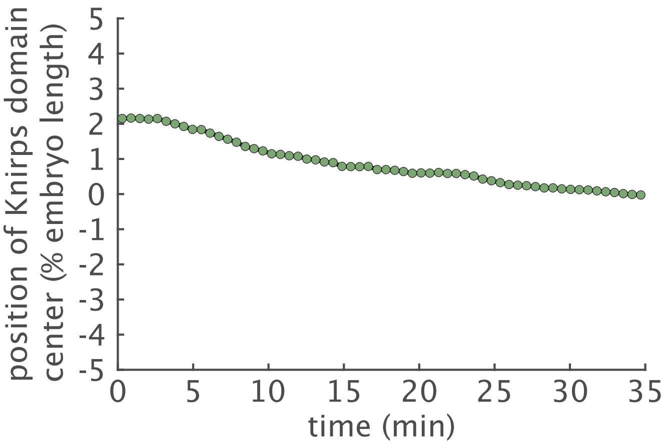

Supplementary Fig. 4: **Temporal dynamics of the Knirps expression center domain.** The graph shows the change in the position of the center of the Knirps expression domain as a function of time. Our analysis reveals that the center of the Knirps domain shifts approximately 2% toward the anterior during nuclear cycle 14, consistent with previous measurements[24].

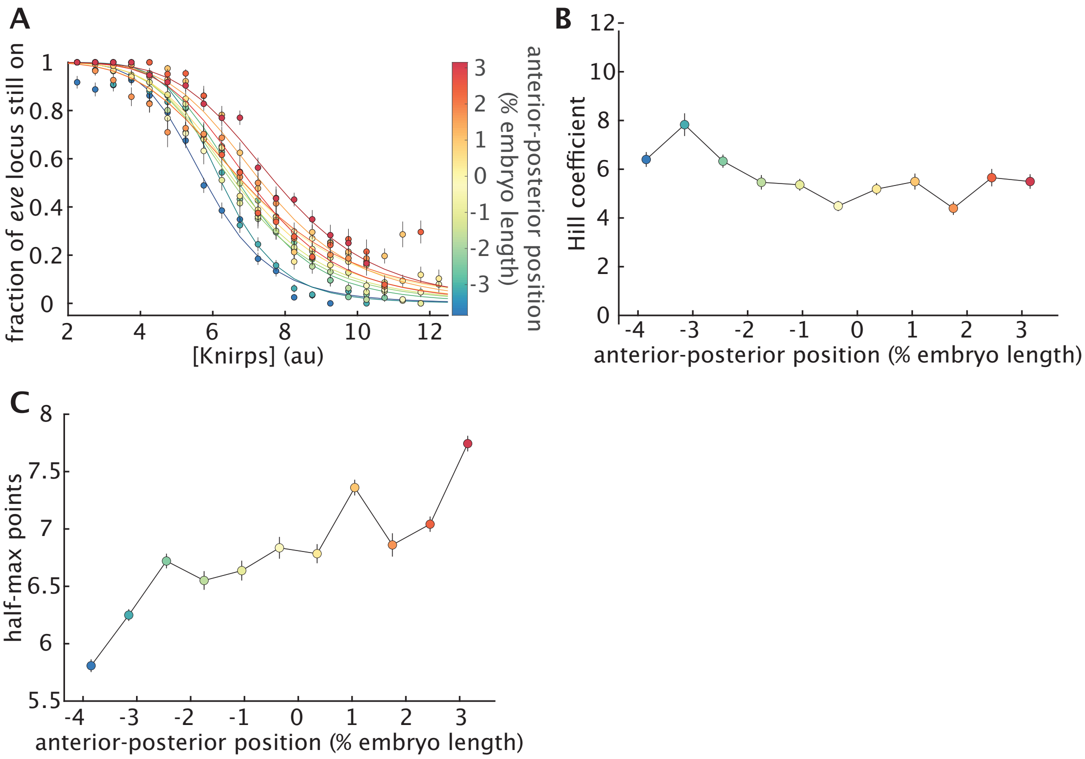

Supplementary Fig. 5: **Analysis of the input-output relationship between Knirps concentration and *eve* 4+6 transcription across the anterior-posterior axis.** (**A**) The shape of the input-output relationship between Knirps concentration and *eve* 4+6 transcription is consistent throughout different regions along the anterior-posterior axis. The input-output functions are calculated over time window of 5 to 40 min. (**B**) The Hill coefficient, which quantifies the responsiveness of *eve* 4+6 transcription to changes in Knirps concentration, is constant across various anterior-posterior positions. This consistency implies that the sharpness of the input-output relationship remains unchanged throughout the axis. (**C**) The Knirps concentration at which half-maximal *eve* 4+6 transcription is induced shifts towards higher values as it approaches the posterior end.

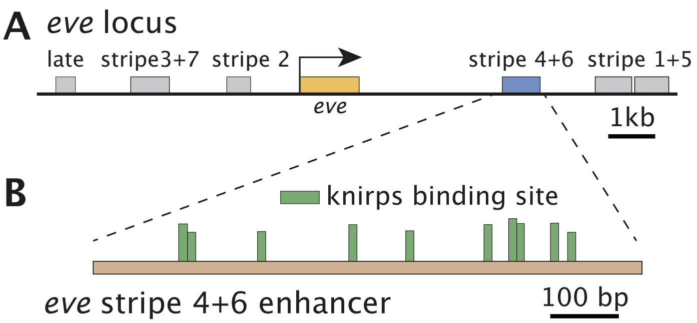

Supplementary Fig. 6: **Predicted Knirps binding sites in the *eve* 4+6 enhancer.** (A) The *eve* 4+6 enhancer is an 800 bp segment from the endogenous *eve* locus. (**B**) Ten Knirps binding sites are predicted within the *eve* 4+6 enhancer using PATSER [25] and Knirps position weight matrices from [26]. Only binding motifs with PATSER scores higher than 3.5 are shown. The bar height of each binding site is proportional to the PATSER score.

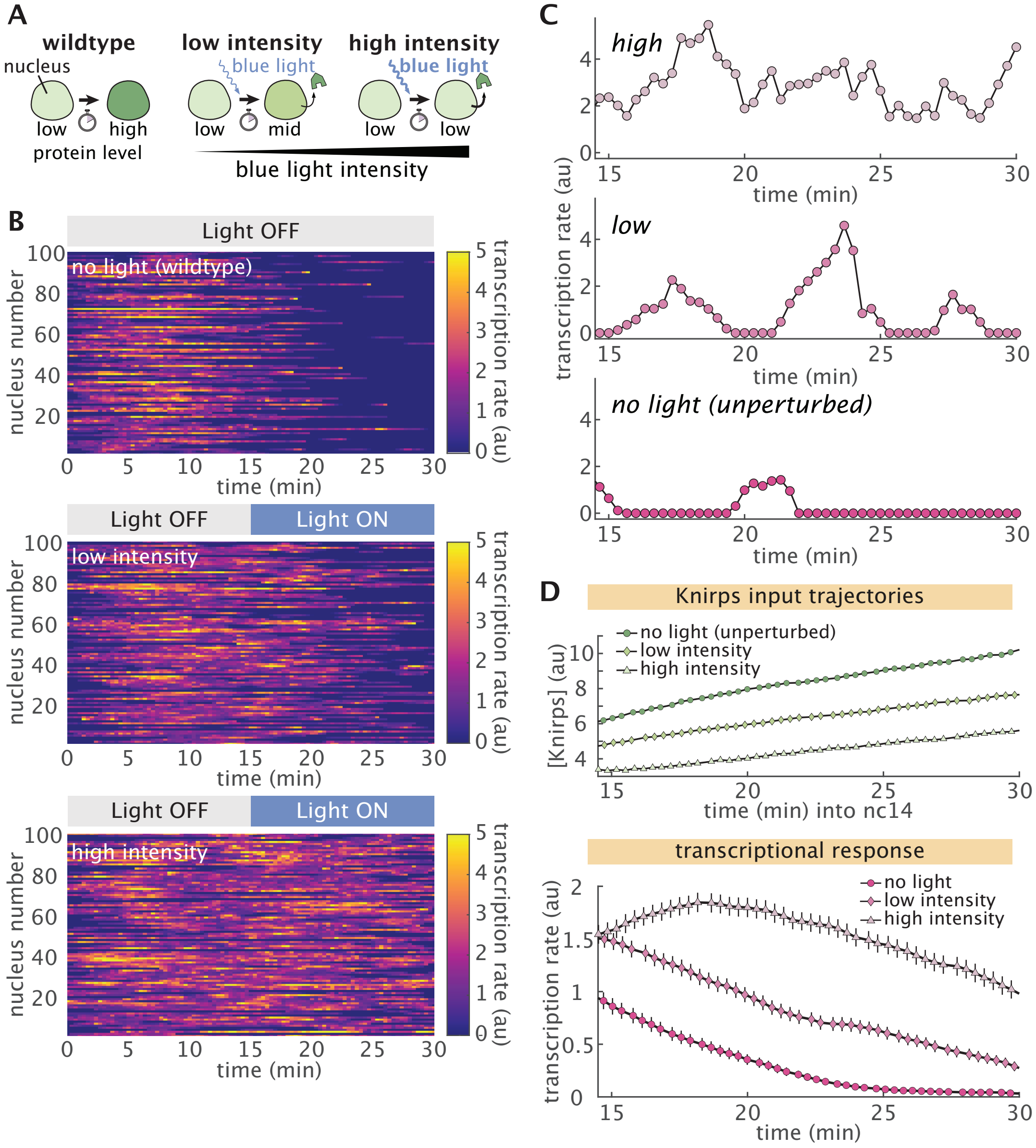

Supplementary Fig. 7: **Repressor titration results in distinct transcriptional dynamics.** (**A**) Optogenetic titration of protein concentration. Cartoon schematics for three different illumination conditions. Left: No illumination results in a negligible export of nuclear Knirps over time (green). Middle: Low dosage of blue light induces weak export of repressor from nuclei. Right: high intensity of blue light results in a strong export of repressor. (**B**) Single-cell traces for embryos with different Knirps export levels show distinct transcriptional dynamics. (**C**) Representative single-cell transcriptional dynamics under different illumination conditions show distinct responses. (**D**) Mean protein (top) and transcription rates (bottom) under different illumination conditions. Averaged over $n = 4$ (no light), $n = 4$ (low intensity) and $n = 3$ (high intensity) embryos. (Error bars in D indicate the bootstrap estimate of the standard error over multiple embryos.)

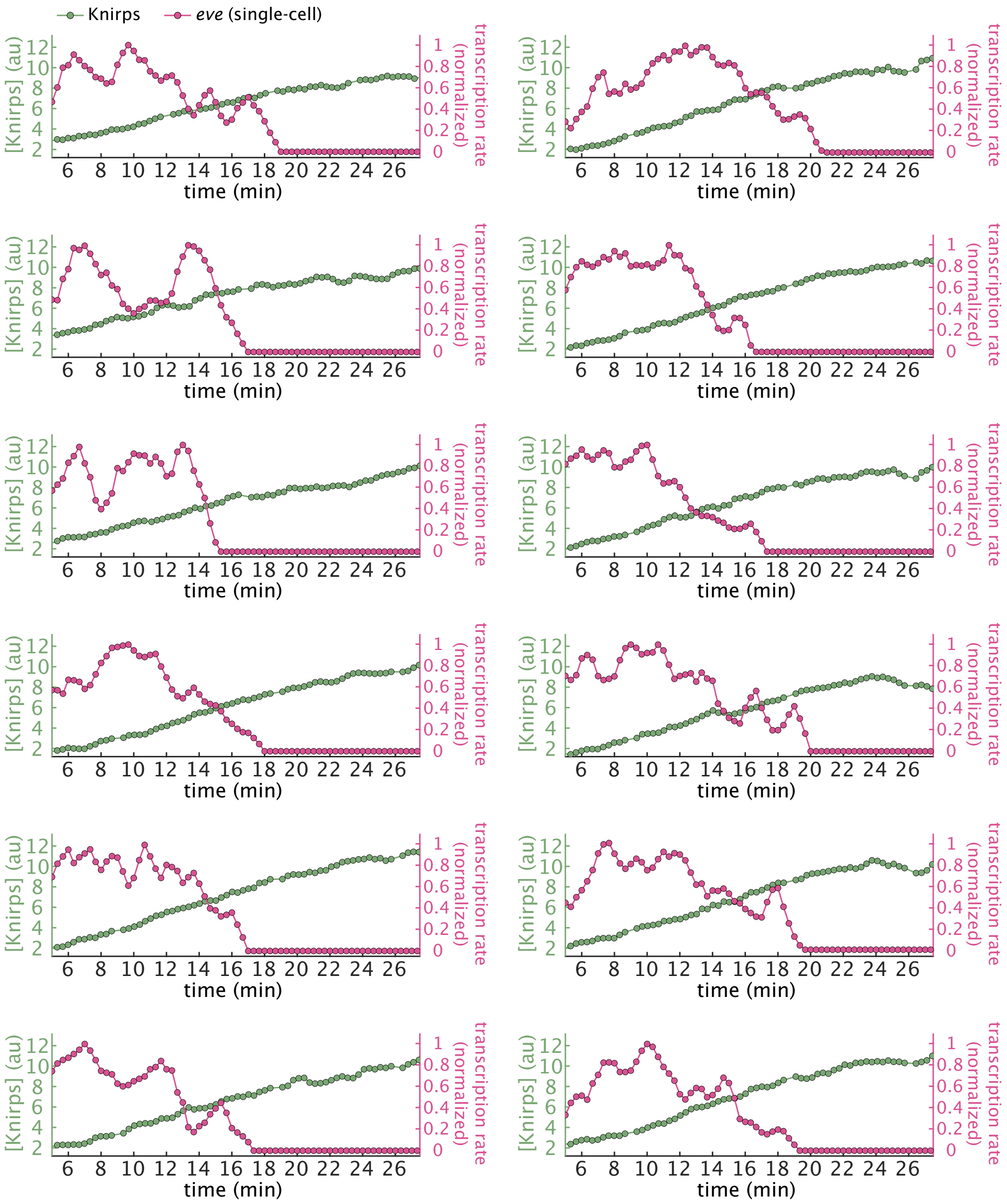

Supplementary Fig. 8: **Example single-cell traces under no illumination.** Single-cell input Knirps and output transcriptional dynamics traces show clear signs of transcriptional bursting, and that repression is switch-like. Traces are normalized by their maximum transcription rate and smoothened using a moving average of 1 minute.

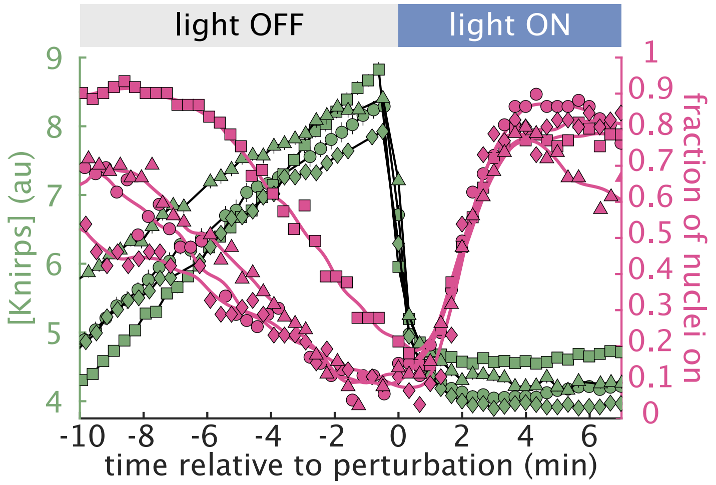

Supplementary Fig. 9: **Response to Knirps perturbations is consistent across multiple embryos.** Plot showing input Knirps concentration and output transcriptional activity for four individual embryos. All embryos display similar responses to Knirps export upon light exposure. Each marker shape corresponds to one embryo. (Error bars indicate the bootstrap estimate of the standard error.)

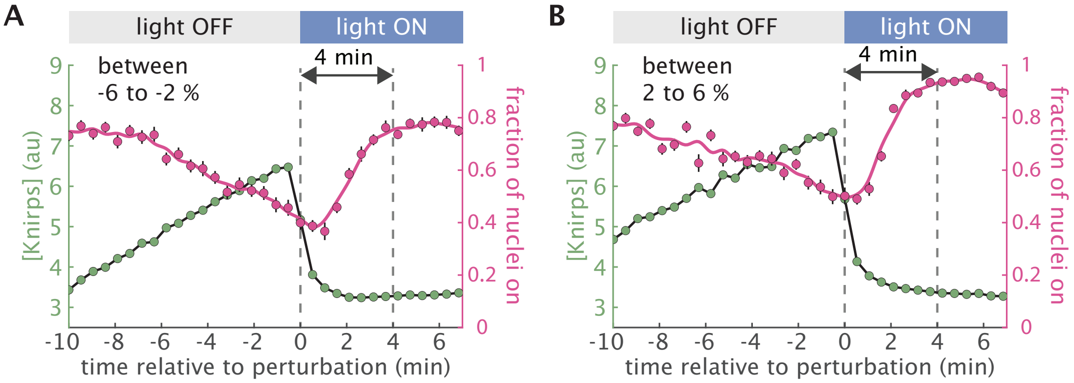

Supplementary Fig. 10: **Reactivation dynamics are consistent across the anterior-posterior axis.** Plot showing input Knirps concentration and output transcriptional activity at two different anterior-posterior positions, between -6 to -2% (A) and between 2 to 6% (B). Both positions display similar reactivation dynamics to Knirps export upon light exposure when compared with wild-type dynamics from Main Text Fig. 3D. (Error bars indicate the bootstrap estimate of the standard error.)

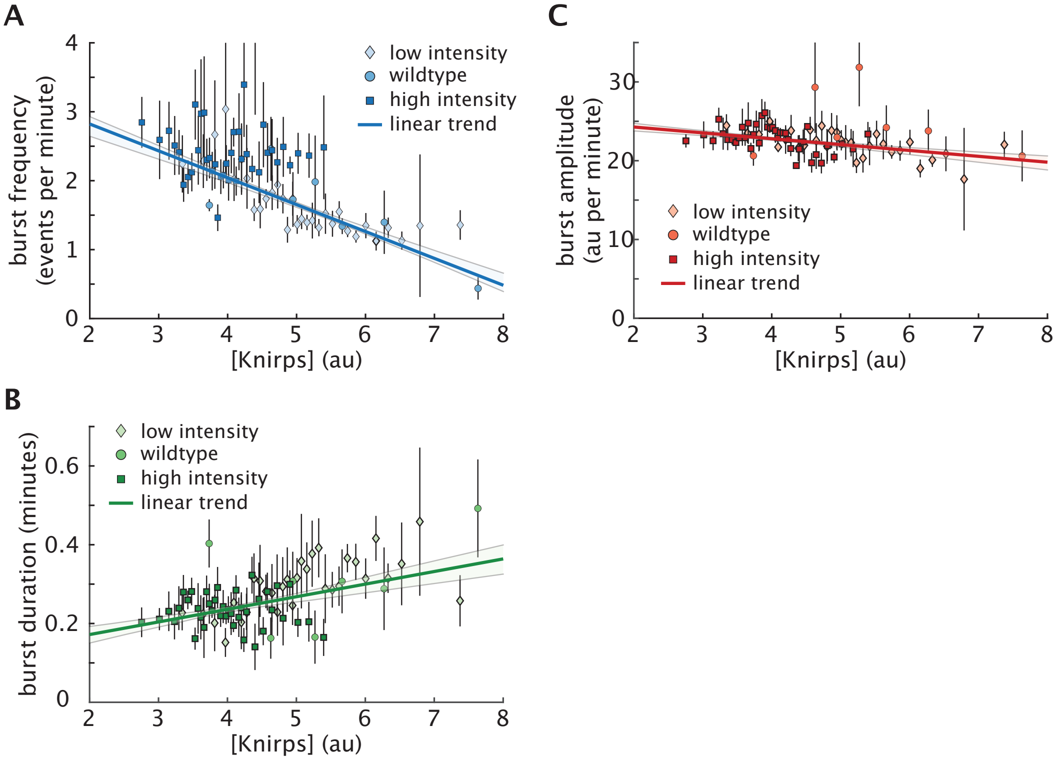

Supplementary Fig. 11: **Full cpHMM inference results of Knirps-regulated transcriptional bursting. (A)** We find that the burst frequency (k_{on}) decreases significantly as a function of Knirps concentration. **(B)** We also find a moderate increase in burst duration ($1/k_{\text{off}}$) with Knirps concentration, **(C)** while burst amplitude (r) remains approximately constant. Lines in A, B and C indicate the best linear fit to data. Circles, diamonds, and squares indicate data points from no light (unperturbed), low illumination, and high illumination experiments, respectively, as described in Main Text Fig. 2B.

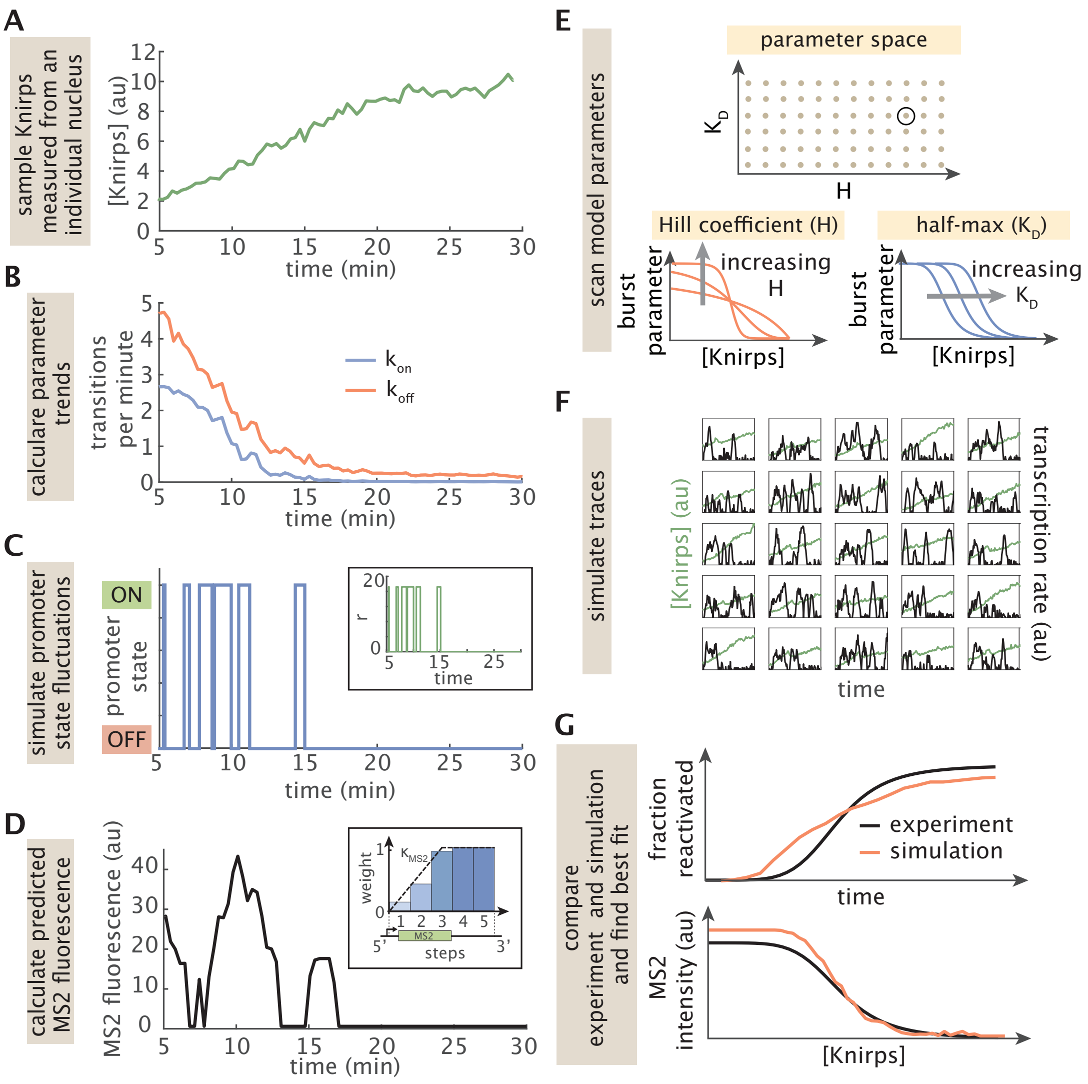

Supplementary Fig. 12: **A computational framework for Knirps-dependent stochastic simulations. (A-D)** Schematic showing process for simulating stochastic transcription time traces. **(A)** We first sample an empirical time trace of Knirps concentration from a nucleus in our live imaging dataset. **(B)** Next, we plug this Knirps trace into the input-output functions for k_{on} (Equation 1) and k_{off} (Equation 2) to generate time-dependent burst parameter trends. **(C)** We then use a discrete implementation of the Gillespie Algorithm to simulate a stochastic time-series of promoter activity that reflects the time-dependent parameter trends. Inset panel shows corresponding initiation rate time series. **(D)** Finally, we use this promoter time series to calculate the predicted MS2 fluorescence at each time point. We assume an initiation rate of 21.5 au when the promoter is in the ON state and a basal rate of 0.6 au when the promoter is OFF. **(E-G)** Schematic illustrating the parameter sweep algorithm. **(E)** We use a simple gridded search to sweep a broad space of values for key parameters in Equations 1 and 2. Cartoon illustrates case for a 2D search for k_{on}-related parameters. In reality, we also scan the analogous k_{off} parameters, leading to a 4D gridded search. For each iteration of the sweep algorithm, we select a new combination of parameters (black circle in top panel). **(F)** Then, we use the process illustrated in A-D to simulate an ensemble of MS2 traces that reflect these parameter values. We generate one simulated MS2 trace for each experimental Knirps trace in our dataset **(G)** Finally, we use these simulated traces to calculate dynamics of the fraction of reactivated and MS2 fluorescence as a function of Knirps concentration for comparison with our experimental results. The mean squared error is used to assess agreement between prediction and experimental data and to identify the set of microscopic parameters that best describes the data.

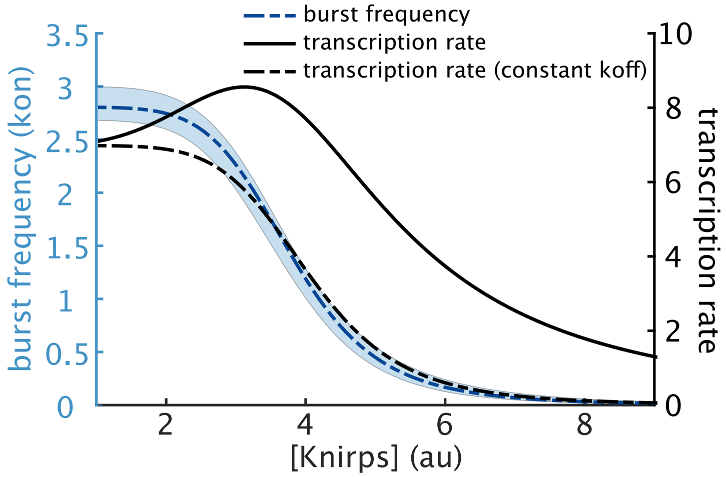

Supplementary Fig. 13: **Examining the offsetting effects of burst duration and burst frequency trends.** The burst parameter analysis presented in the main text indicated that Knirps represses *eve* 4+6 by down-regulating the burst frequency (Main Text Fig. 4C, left panel, and F). Our results also indicated a more moderate *increase* in burst duration at higher Knirps concentrations (center panel of Main Text Fig. 4C). Because the rate of transcription depends on both the burst frequency (k_{on}) and the burst duration ($1/k_{\mathrm{off}}$), it is less sensitive to Knirps concentration than k_{on} alone. The above plot compares our inferred burst frequency vs. Knirps trend shown in Main Text Fig. 4F (dashed blue curve) to the predicted rate of transcription with variable (solid black trend) and fixed (dashed black trend) burst duration values. We see that holding burst duration fixed results in a transcription rate that closely mirrors the burst frequency trend, whereas allowing burst duration to increase (following the trend shown in Supplementary Fig. 14C) shifts the predicted rate curve rightward, in accordance with the actual sensitivity we observe in our data for the mean transcription rate (see e.g. Main Text Fig. 4G). Thus, while the burst duration does not contribute directly to *eve* 4+6 repression, it is predicted to impact the sensitivity of the gene locus to Knirps concentration.

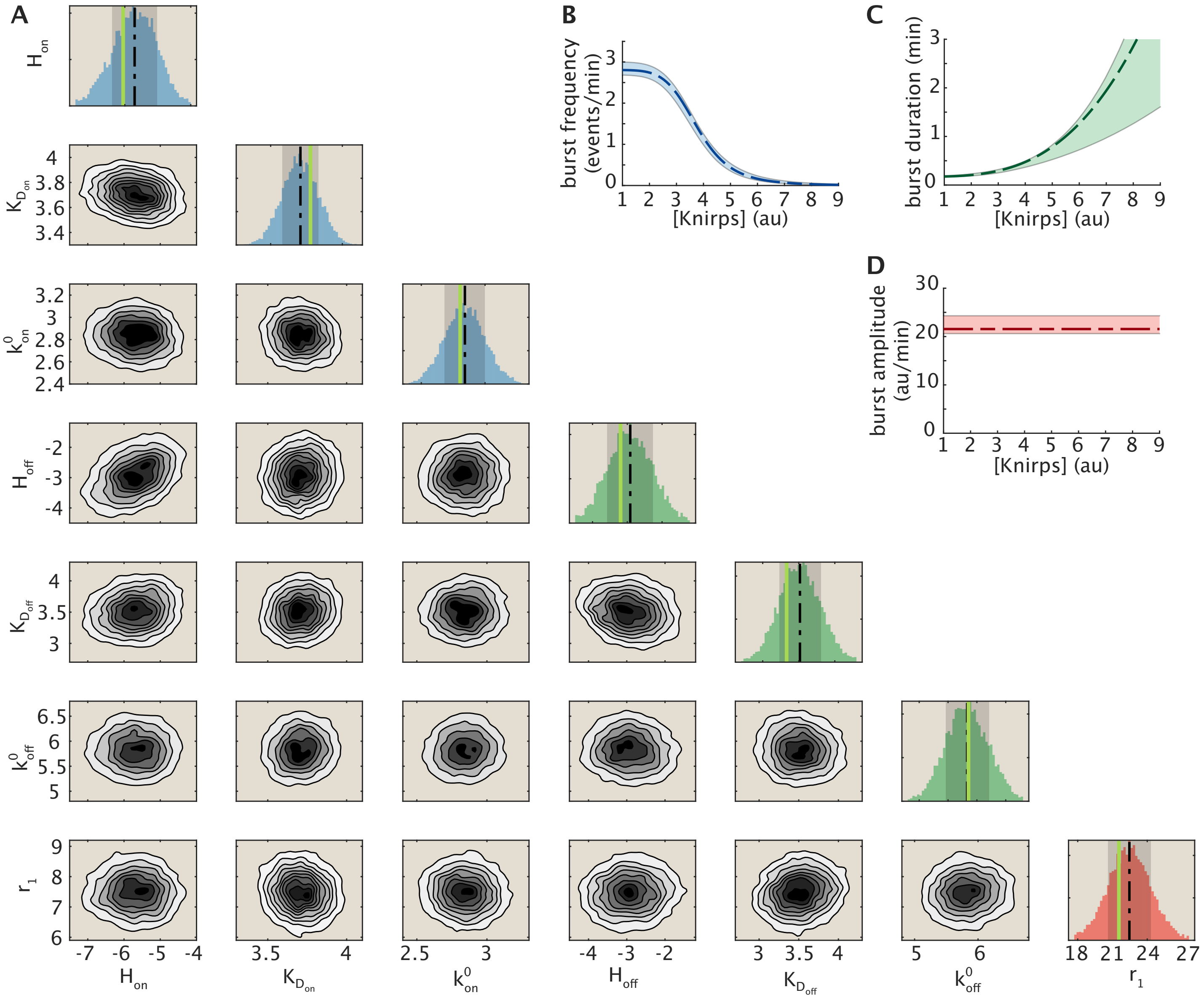

Supplementary Fig. 14: **Full MCMC results for stochastic input-output model parameters.** (**A**) Univariate and bivariate density plots. Vertical green lines in histograms indicate the mean parameter value taken across the 25 best-fitting model realizations. Dashed black lines indicate average parameter values taken across all MCMC samples; i.e. the full distribution shown in each histogram. Shaded regions in histograms indicate 1 standard deviation above and below the mean. (**B**) Inferred trends for the burst frequency (k_{on}), **(C)** burst duration ($1/k_{\text{off}}$) and **(D)** burst amplitude (r_1). k_{off} was modeled as a Hill function of Knirps (see Equation 2) and r was assumed to be invariant relative to Knirps concentration.

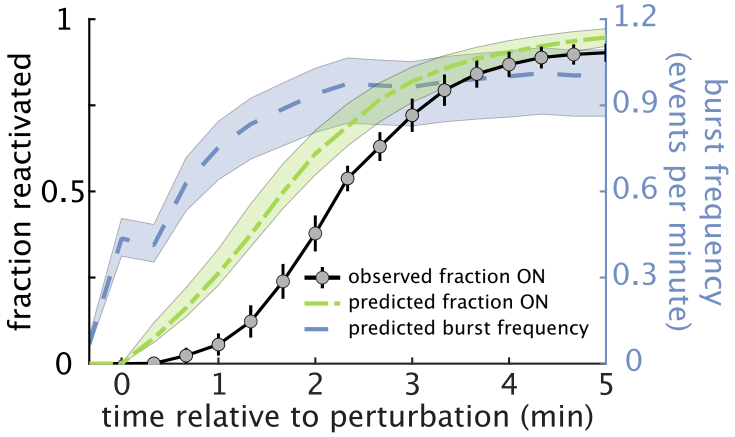

Supplementary Fig. 15: **Model predictions for *eve*4+6 reactivation dynamics following Knirps export.** Blue curve shows the predicted recovery of burst frequency (calculated from Equation 1) following the optogenetic perturbation of Knirps. The green curve indicates the corresponding cumulative fraction of loci that are predicted to have reentered the ON state as a function of time since the perturbation. Black curve is identical to the one shown in Main Text Fig. 4H and corresponds to the measured fraction of loci that have reentered the ON state. We observe a lag between the cumulative fraction of ON loci and the experimentally observed fraction because recently reactivated gene loci typically require multiple time steps to accumulate sufficient fluorescent MS2 signal in order to be experimentally detected.

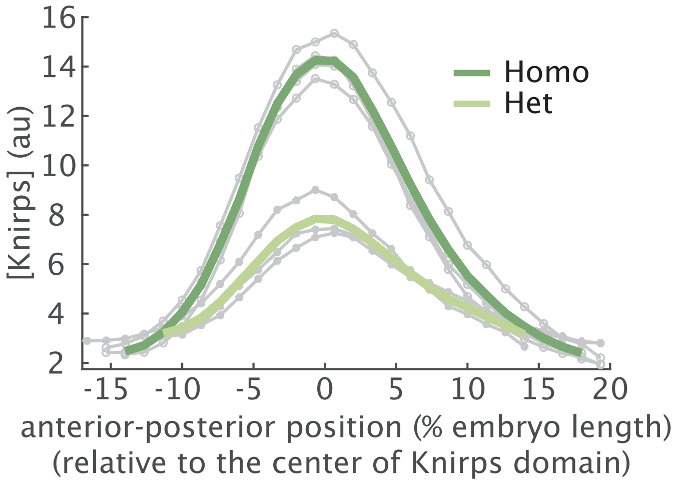

Supplementary Fig. 16: **Distinguishing homozygous from heterozygous embryos.** Homozygous embryos ($n = 4$) can be easily distinguished from heterozygous embryos ($n = 3$) by comparing Knirps concentration at 30 min into nc14.

Supplementary Tables

Supplementary Table 1: **List of plasmids used in this study.**

**Name**	**Function**
pCasper-vasaPr-EYFP	P-element insertion plasmid for vasa promoter driven EYFP
pBPhi-eve4+6-evePr-MS2-Yellow	*eve* 4+6 reporter
pHD-Kni-LlamaTag-LEXY-dsRed	Donor plasmid for Knirps-LlamaTag-LEXY CRISPR knock-in fusion
pU6-3-gRNA-Knirps-1	guide RNA 1 for Knirps-LlamaTag-LEXY CRISPR knock-in fusion
pU6-3-gRNA-Knirps-2	guide RNA 2 for Knirps-LlamaTag-LEXY CRISPR knock-in fusion

Supplementary Table 2: **List of fly lines used in this study.**

**Genotype**	**Usage**
yw; vasa-EYFP; +	Maternally deposit ubiquitous EYFP
yw; +; MCP-mCherry/TM3,Sb	Maternally deposit MCP-mCherry protein
yw; eve4+6-evePr-MS2-Yellow; +	MS2 reporter for *eve* 4+6 enhancer
yw; +; Kni-LlamaTag-LEXY	CRISPR knock-in of LlamaTag and LEXY at Knirps C-terminal
yw; vasa-EYFP; MCP-mCherry/TM3,Sb	Maternally deposit both ubiquitous EYFP and MCP-mCherry proteins
yw; vasa-EYFP; Kni-LlamaTag-LEXY	Maternally deposit ubiquitous EYFP, and expresses Knirps protein labeled with LlamaTag and LEXY
yw; eve4+6-evePr-MS2-Yellow; Kni-LlamaTag-LEXY	MS2 reporter for *eve* 4+6 enhancer with endogenous *knirps* locus labeled with LlamaTag and LEXY

Supplementary Table 3: **List of parameter ranges used for parameter sweeps.** Brackets denote inclusive ranges. Parameters with a single value appearing in the "range" column were held fixed during the sweeps. Parameters with two values were sampled at 15 equally spaced points bounded by the values indicated in the brackets.

**Parameter**	**Range**
burst frequency Hill Coefficient (H_{ON})	[3.15, 12.6]
burst frequency half-maximum ($K_{D_{\text{ON}}}$)	[2.5, 10.2] (au)
max burst frequency (k^0_{on})	2.85 (events per min)
off rate Hill Coefficient (H_{OFF})	[0, 4]
off rate half-maximum ($K_{D_{\text{ON}}}$)	[2, 6] (au)
max off rate (k^0_{off})	5.81 (events per min)
ON state initiation rate (r_1)	22.76 (au per min)
OFF state initiation rate (r_0)	0.6 (au per min)

Supplementary Table 4: **List of parameter priors used for MCMC sampling.**

**Parameter**	**Prior distribution**
burst frequency Hill Coefficient (H_{ON})	$\mathcal{N}(5.7, 0.8)$
burst frequency half-maximum ($K_{D_{\text{ON}}}$)	$\mathcal{N}(3.7, 0.15)$ (au)
max burst frequency (k^0_{on})	$\mathcal{N}(2.84, 0.17)$ (events per min)
off rate Hill Coefficient (H_{OFF})	$\mathcal{N}(3.1, 0.8)$
off rate half-maximum ($K_{D_{\text{ON}}}$)	$\mathcal{N}(3.5, 0.3)$ (au)
max off rate (k^0_{off})	$\mathcal{N}(5.8, 0.4)$ (events per min)
initiation rate (r_1)	$\mathcal{N}(22.8, 2.1)$ (au per min)

Supplementary References

1. Gillespie, D. T. Exact stochastic simulation of coupled chemical reactions. *Journal of Physical Chemistry* **81,** 2340–2361 (1977).
2. Lammers, N. C. *et al.* Multimodal transcriptional control of pattern formation in embryonic development. *PNAS* **117,** 836–847 (2020).
3. Geyer, C. J. Practical Markov Chain Monte Carlo. *Statistical Science,* 473–483 (1992).
4. Metropolis, N., Rosenbluth, A. W., Rosenbluth, M. N., Teller, A. H. & Teller, E. Equation of state calculations by fast computing machines. *The Journal of Chemical Physics* **21,** 1087–1092 (1953).
5. Robert, C. P. & Casella, G. in *Monte Carlo statistical methods* 267–320 (Springer, 2004).
6. Lammers, N. C., Kim, Y. J., Zhao, J. & Garcia, H. G. A matter of time: Using dynamics and theory to uncover mechanisms of transcriptional bursting. *Current Opinion in Cell Biology* **67,** 147–157 (2020).
7. Niopek, D., Wehler, P., Roensch, J., Eils, R. & Di Ventura, B. Optogenetic control of nuclear protein export. *Nature Communications* **7,** 1–9 (2016).
8. Kögler, A. C. *et al.* Extremely rapid and reversible optogenetic perturbation of nuclear proteins in living embryos. *Developmental Cell* **56,** 2348–2363 (2021).
9. Toh, P. J. Y. *et al.* Optogenetic control of YAP cellular localisation and function. *EMBO Reports,* e54401 (2022).
10. Singh, A. P. *et al.* Optogenetic control of the Bicoid morphogen reveals fast and slow modes of gap gene regulation. *Cell Reports* **38,** 110543 (2022).
11. Meyer, K., Lammers, N. C., Bugaj, L. J., Garcia, H. G. & Weiner, O. D. Optogenetic control of YAP reveals a dynamic communication code for stem cell fate and proliferation. *Nature Communications* **14** (Dec. 2023).
12. Wang, X., He, L., Wu, Y. I., Hahn, K. M. & Montell, D. J. Light-mediated activation reveals a key role for Rac in collective guidance of cell movement *in vivo*. *Nature Cell Biology* **12,** 591–597 (2010).
13. Izquierdo, E., Quinkler, T. & De Renzis, S. Guided morphogenesis through optogenetic activation of Rho signalling during early *Drosophila* embryogenesis. *Nature Communications* **9,** 2366 (2018).
14. Johnson, H. E. *et al.* The spatiotemporal limits of developmental Erk signaling. *Developmental Cell* **40,** 185–192 (2017).
15. Johnson, H. E., Djabrayan, N. J., Shvartsman, S. Y. & Toettcher, J. E. Optogenetic rescue of a patterning mutant. *Current Biology* **30,** 3414–3424 (2020).
16. Keenan, S. E. *et al.* Rapid dynamics of signal-dependent transcriptional repression by Capicua. *Developmental Cell* **52,** 794–801 (2020).
17. Patel, A. L. *et al.* Capicua is a fast-acting transcriptional brake. *Current Biology* **31,** 3639–3647 (2021).

18. Sako, K. *et al.* Optogenetic control of Nodal signaling reveals a temporal pattern of Nodal signaling regulating cell fate specification during gastrulation. *Cell Reports* **16,** 866–877 (2016).

19. Chan, Y. B., Alekseyenko, O. V. & Kravitz, E. A. Optogenetic control of gene expression in *Drosophila*. *PLoS ONE* **10,** e0138181 (2015).

20. Huang, A., Amourda, C., Zhang, S., Tolwinski, N. S. & Saunders, T. E. Decoding temporal interpretation of the morphogen Bicoid in the early *Drosophila* embryo. *eLife* **6,** e26258 (2017).

21. McDaniel, S. L. *et al.* Continued activity of the pioneer factor Zelda is required to drive zygotic genome activation. *Molecular Cell* **74,** 185–195 (2019).

22. Lammers, N. C., Flamholz, A. I. & Garcia, H. G. Competing constraints shape the nonequilibrium limits of cellular decision-making. en. *Proc. Natl. Acad. Sci. U. S. A.* **120,** e2211203120 (Mar. 2023).

23. Berrocal, A., Lammers, N., Garcia, H. G. & Eisen, M. B. Kinetic sculpting of the seven stripes of the *Drosophila even-skipped* gene. *eLife* **9,** e61635 (2020).

24. Jaeger, J. *et al.* Dynamic control of positional information in the early *Drosophila* embryo. *Nature* **430,** 368–371 (2004).

25. Hertz, G. Z. & Stormo, G. D. Identifying DNA and protein patterns with statistically significant alignments of multiple sequences. *Bioinformatics* **15,** 563–577 (1999).

26. Estrada, J., Ruiz-Herrero, T., Scholes, C., Wunderlich, Z. & DePace, A. H. SiteOut: An online tool to design binding site-free DNA sequences. *PLoS ONE* **11,** e0151740 (2016).
