# Supplementary material for: Optogenetic dissection of transcriptional repression in a multicellular organism
Source: Nat Commun. 2024 Oct 26;15:9263. doi: 10.1038/s41467-024-53539-0 (PMC11513125; doi:10.1038/s41467-024-53539-0)
Supplement: Supplementary file 3 — Description of Additional Supplementary Files [file 41467_2024_53539_MOESM3_ESM.pdf]

Description of Additional Supplementary Files

File Name: Supplementary Movie 1
Description: Full movie for repression without perturbation. Knirps concentration is indicated in green. Active eve 4+6 loci appear in magenta. Timestamp indicates minutes since the start of nuclear cycle 14.

File Name: Supplementary Movie 2
Description: Full movie demonstrating optogenetic manipulation of protein concentration. Knirps concentration is indicated in green. Timestamp indicates time in minutes relative to the optogenetic perturbation.

File Name: Supplementary Movie 3
Description: Full movie demonstrating optogenetic titration of protein concentration.} Panels correspond to the three illumination conditions illustrated in Main Text Fig. 2B. Knirps concentration is indicated in green. Active eve 4+6 loci appear in magenta. Timestamp indicates minutes since the start of nuclear cycle 14.

File Name: Supplementary Movie 4
Description: Full movie showing optogenetic export of repressor protein. Knirps concentration is indicated in green. Active eve 4+6 loci appear in magenta. Timestamp indicates time in minutes relative to the perturbation.
